#  Phospholipid Scramblases TMEM16F and Xkr8 regulate distinct features of Phosphatidylserine (PS) externalization and immune regulation in the tumor microenvironment to regulate tumor growth 

**DOI:** 10.1101/2025.04.17.649445

**Published:** 2025-04-18

**Authors:** Varsha Gadiyar, Rachael Pulica, Ahmed Aquib, James A. Tranos, Christopher Varsanyi, Luis Fernandez Almansa, Lawrence Gaspers, Mariana De Lorenzo, Sergei V Kotenko, Sushil Tripathi, Roger W. Howell, Alok Choudhary, David C. Calianese, Raymond B. Birge

## Abstract

The phospholipid scramblases Xkr8 and TMEM16F externalize phosphatidylserine (PS) on cells by distinct molecular mechanisms. Xkr8, a caspase-activated scramblase, is activated by caspase-mediated proteolytic cleavage, and in synergy with caspase-mediated inactivation of P4-type ATP-dependent flippases, results in the irreversible externalization of PS on the dying cells and an “eat-me” signal for efferocytosis. In contrast, TMEM16F is a calcium activated scramblase that reversibly externalizes PS on viable cells via the transient increase in intracellular calcium on activated or growth factor stimulated cells. By contrast to the abovementioned homeostatic mechanisms of PS externalization under physiological conditions, PS becomes constitutively externalized in the tumor microenvironment (TME) in many solid tumor types by a complex mechanistic, posited both via the high apoptotic indexes of tumors, but also by the prolonged oncogenic and metabolic stresses that occur in the TME. Such chronic and persistent PS externalization in the TME has been linked to host immune evasion and the tonic interactions of PS with inhibitory PS receptors such as TAM (Tyro3, Axl, Mertk) and TIM (T cell/transmembrane, immunoglobulin, and mucin) family receptors. Here, in an effort to better understand the contributions of apoptotic vs live cell PS-externalization with respect to tumorigenesis and immune evasion, we employed an E0771 luminal B breast cancer orthotopic *in vivo* model and genetically ablated Xkr8 and TMEM16F using CRISPR/Cas9. While neither the knockout of Xkr8 nor TMEM16F showed defects in cell intrinsic properties related to cell growth, tumor sphere formation, cell migration, and growth factor signaling, both knockouts suppressed tumorigenicity in immune-competent mice, but not in NOD/SCID or RAG deficient immune-deficient strains. Mechanistically, at the cell biological level, Xkr8 knockout suppressed macrophage-mediated efferocytosis, and TMEM16F knockout suppressed ER stress/calcium-induced PS externalization. Our data support an emerging idea in immune-oncology and immunotherapy that constitutive PS externalization, mediated by the activation of scramblases on tumor cells, can support immune evasion in the tumor microenvironment thereby linking a combination of apoptosis/efferocytosis and oncogenic stress involving calcium dysregulation the contribute to PS-mediated immune escape and cancer progression.

## Full Text Availability

The license terms selected by the author(s) for this preprint version do not permit archiving in PMC. The full text is available from the preprint server.

