# Supplementary material for: Phospholipid Scramblases TMEM16F and Xkr8 mediate distinct features of Phosphatidylserine (PS) externalization and immune suppression to promote tumor growth
Source: bioRxiv. 2025 Jun 23:2025.04.17.649445. Originally published 2025 Apr 18. Preprint. [Version 2] doi: 10.1101/2025.04.17.649445 (PMC12087989; doi:10.1101/2025.04.17.649445)
Supplement: Supplement 1 — (S.1.A) Kaplan-Meier survival analysis of breast cancer patient data, showing a negative correlation between Xkr8 gene expression and patient survival, with higher Xkr8 levels associated with poorer survival. (1.B) Kaplan-Meier survival analysis of breast cancer patients, comparing low and high TMEM16F gene expression. Patients with low TMEM16F expression exhibited better survival compared to those with high TMEM16F expression. (S.2.A.) Surveyor assay showing a successful KO of Xkr8 gene in EO771 cells. (S.2.B.) p-Akt signaling upon treatment of scramblase KO cells with Gas6 shows no significant difference in their intrinsic Axl-Gas6 signaling capabilities. (S.2.C.) Quantification of metastatic nodules in the lung from NSG mice injected with scramblase KO tumors. (S.2.D.) Incucyte imaging quantifying cell death upon different treatments with the Cytotox dye shows that glucose deprivation and MG132 are not toxic to cells at earlier time points. (S.2.E.) Quantification of intracellular calcium and annexin V staining on EO771 cells treated with calcium ionophore shows an early spike with calcium which subsides with time. (S.2.F.) Hypoxic conditions for 24 hours with 2% O2 did not induce live cell PS externalization in EO771 cells. [file media-1.pptx]

## Slide 1
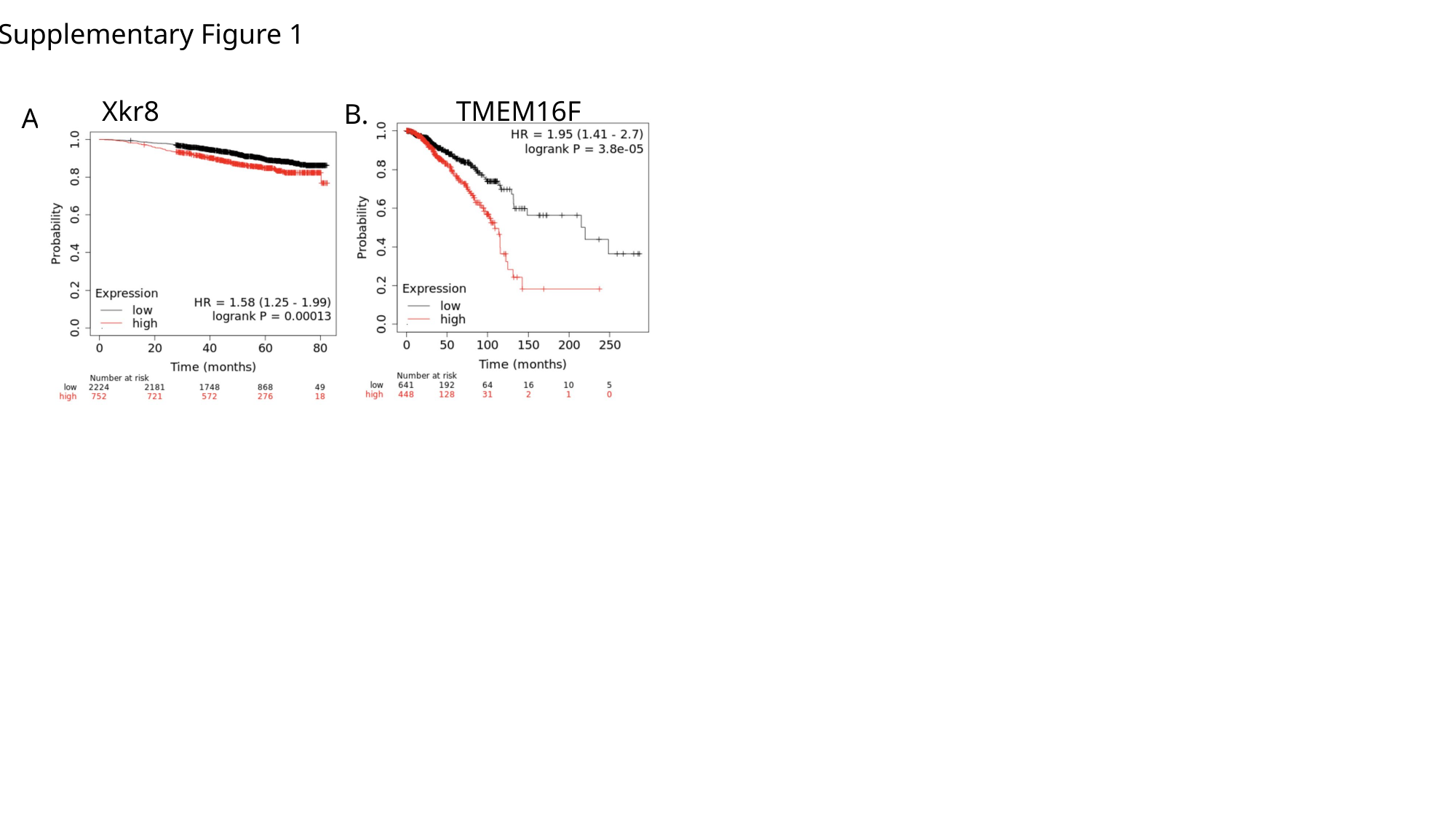

Supplementary Figure 1
Xkr8
TMEM16F
B.
A.

## Slide 2
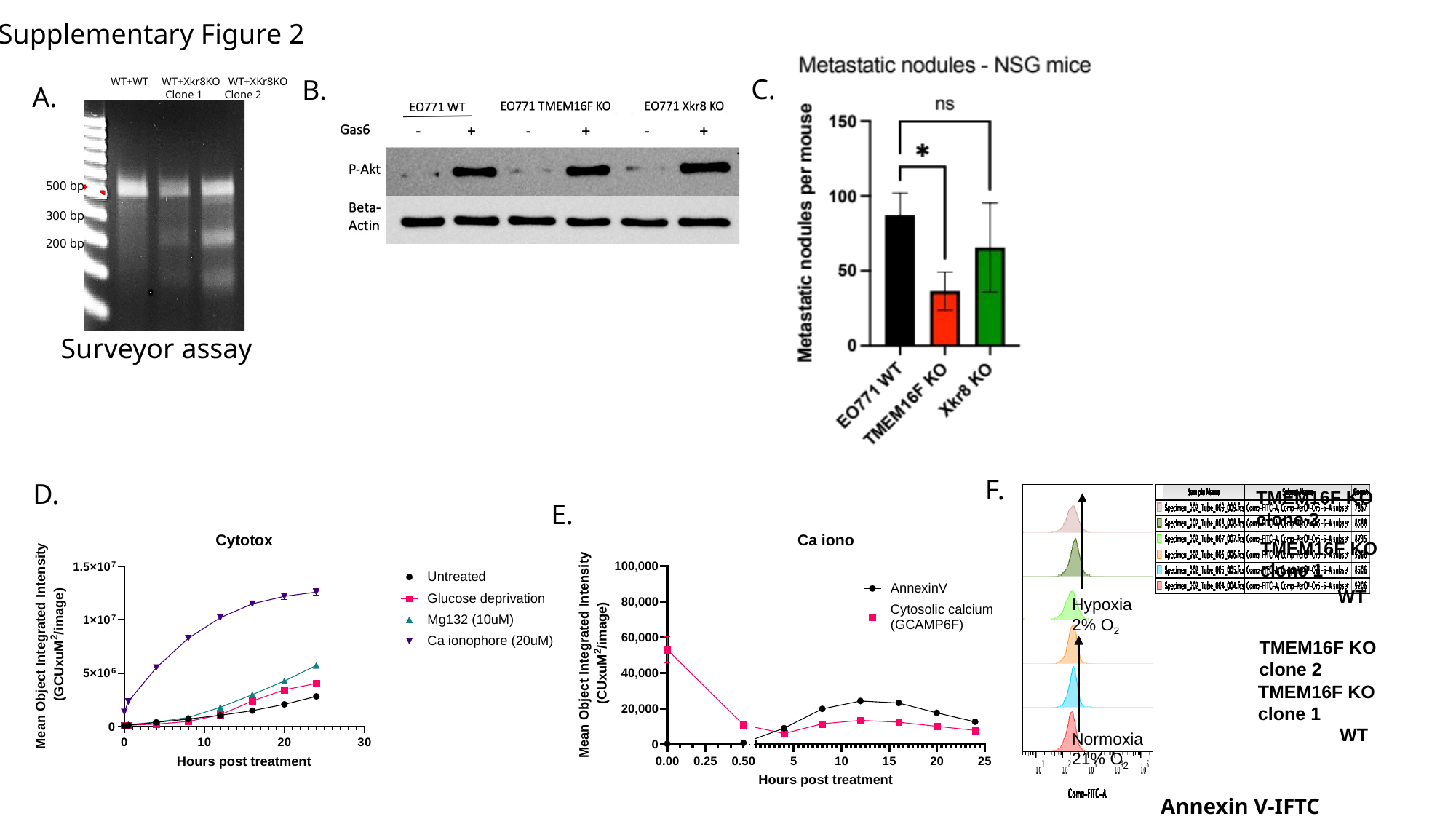

Supplementary Figure 2
C.
B.
WT+WT WT+Xkr8KO WT+XKr8KO
 Clone 1 Clone 2
500 bp
300 bp
200 bp
A.
Surveyor assay
F.
D.
TMEM16F KO clone 2
TMEM16F KO clone 1
WT
Hypoxia 2% O2
TMEM16F KO clone 2
TMEM16F KO clone 1
WT
Normoxia 21% O2
Annexin V-IFTC
E.
